# Convolutional neural network in rice disease recognition: accuracy, speed and lightweight

**DOI:** 10.3389/fpls.2023.1269371

**Published:** 2023-11-01

**Authors:** Hongwei Ning, Sheng Liu, Qifei Zhu, Teng Zhou

**Affiliations:** ^1^ College of Information and Network Engineering, Anhui Science and Technology University, Bengbu, Anhui, China; ^2^ Information Network Security College, Yunnan Police College, Kunming, Yunnan, China; ^3^ Mechanical and Electrical Engineering College, Hainan University, Haikou, Hainan, China

**Keywords:** rice disease, convolutional neural network, lightweight, model compression, convolution operation

## Abstract

There are many rice diseases, which have very serious negative effects on rice growth and final yield. It is very important to identify the categories of rice diseases and control them. In the past, the identification of rice disease types was completely dependent on manual work, which required a high level of human experience. But the method often could not achieve the desired effect, and was difficult to popularize on a large scale. Convolutional neural networks are good at extracting localized features from input data, converting low-level shape and texture features into high-level semantic features. Models trained by convolutional neural network technology based on existing data can extract common features of data and make the framework have generalization ability. Applying ensemble learning or transfer learning techniques to convolutional neural network can further improve the performance of the model. In recent years, convolutional neural network technology has been applied to the automatic recognition of rice diseases, which reduces the manpower burden and ensures the accuracy of recognition. In this paper, the applications of convolutional neural network technology in rice disease recognition are summarized, and the fruitful achievements in rice disease recognition accuracy, speed, and mobile device deployment are described. This paper also elaborates on the lightweighting of convolutional neural networks for real-time applications as well as mobile deployments, and the various improvements in the dataset and model structure to enhance the model recognition performance.

## 1 Introduction

In recent years, rice planting has developed quickly and the mechanization degree has been gradually improved (Kabir et al., 2021). However, rice disease has always been a huge obstacle to the further development of rice planting (Azim et al., 2021). The disease has always been an important factor restricting rice growth, high and stable yield. Rice disease affects the total grain loss of up to 10% to 30% in the world every year (Agrawal and Agrawal, 2020). Therefore, rapid and accurate identification of rice diseases is very important for ensuring rice production and maintaining global food security (Daniya and Vigneshwari, 2022a).

The traditional rice disease recognition generally relies on the experience accumulation of farmers in the actual production process (Yakkundimath et al., 2022). This method has high professional requirements for practitioners, consumes a lot of workforce and costs. But the judgment outcomes are highly biased, with large errors even (Sony, 2019). It is challenging to achieve accurate disease identification, simple to lose the best time for disease preclusion and control. At the same time, it is difficult to meet current needs for real-time monitoring and prediction of a wide range of diseases (Chen J. et al., 2020). Therefore, it is significant to investigate automatic recognition methods of rice typical diseases for early detection, diagnosis, and treatment of rice diseases to reduce loss and increase yield (Jadhav et al., 2021; Jiang et al., 2021).

With the enhancement of computing power of computer hardware and the explosive growth of data, deep learning technology has achieved very good results and been broadly applied in many areas such as speech recognition, image processing, and natural language processing (Domingues et al., 2022; Ning et al., 2022). The convolutional neural network is a very representative deep learning technology (Priyangka and Kumara, 2021). Its performances in computer vision missions such as image semantic segmentation, object detection, and classification recognition are far superior to other traditional methods. It has been extensively used in face recognition, automatic driving, and other engineering fields (Brahimi et al., 2018; Sethy et al., 2020a).

**Figure 1:**
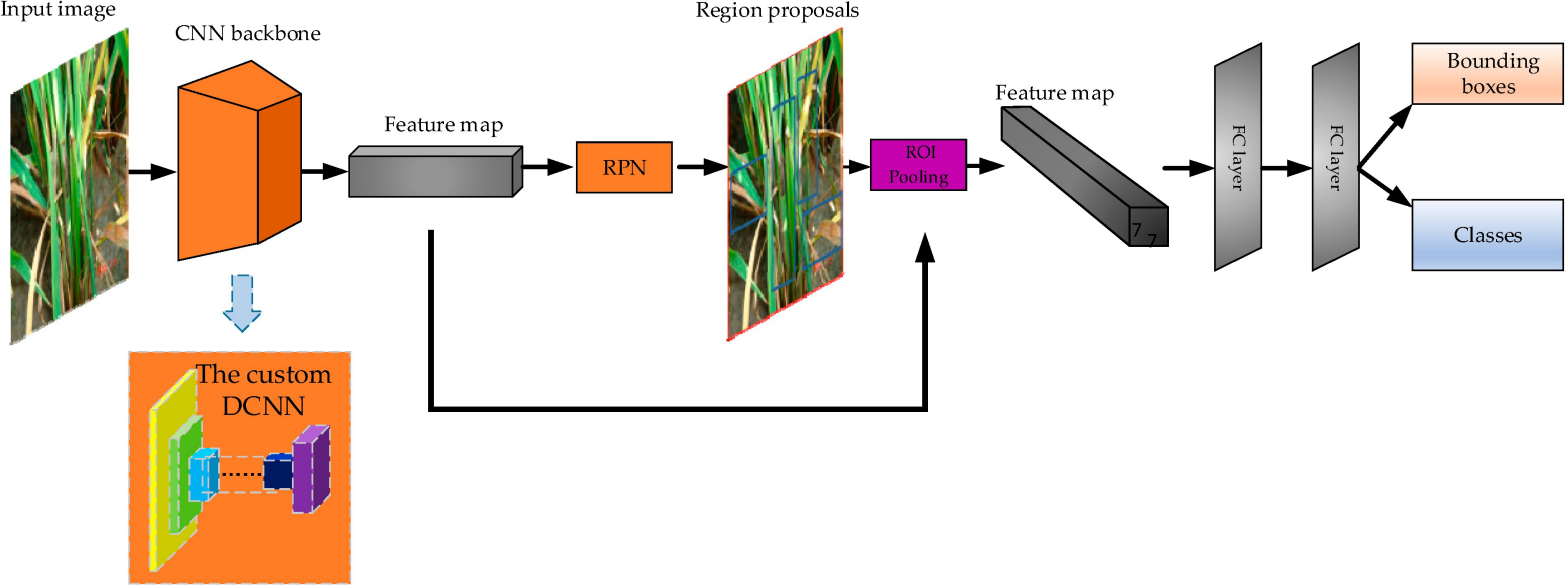
Rice disease detection model diagram. (Reprinted from ref. (Li et al., 2020) under the terms of the Creative Commons CC-BY license).

In terms of rice disease recognition, convolutional neural networks have also obtained very good results and been widely utilized (Li et al., 2020; Sharma et al., 2020) (**Figure 1**). In **Figure 1**, the CNN backbone is a common convolutional neural network such as ResNet50,VGG16 etc. which is mainly responsible for extracting various features from images. The RPN searches for candidate regions where rice diseases may be present from the extracted features by traversing them at a time. The ROI pooling converts all candidate regions into a format that is uniform in length and width. The final fully connected layer unifies all the candidate regions and outputs the type of rice disease and its location in the image. With deepening application, the recognition of rice disease has put forward new requirements for convolutional neural networks. In the paper, the achievements of convolutional neural networks in rice disease recognition in recent years are summarized, and the recognition accuracy optimization, recognition speed improvement, and lightweight revision are discussed respectively. The contributions of the paper are the following:

- Theory of convolutional neural networks is explained detailed, especially various measures that could reduce the complexity of convolutional neural networks.
- Methods that can improve the accuracy of rice disease identification based on convolutional neural networks are summarized.
- Existing research results for speeding up convolutional neural networks to identify rice diseases are summarized.
- The deployment of convolutional neural networks to mobile devices for real-time rice disease identification is summarized.
- The limitations of rice disease recognition based on convolutional neural networks are given and the future research priorities are envisioned.

The structure of this paper is organized as follows: the first part is the introduction; the second part discusses the basic principles and lightweight measures of convolutional neural networks; the third part summarizes the method of convolutional neural network to improve the recognition accuracy in the process of rice disease recognition; in the fourth part, the measures to deploy convolutional neural networks on mobile devices after lightweight revision in the process of rice disease recognition are discussed; the fifth part summarizes the approaches to optimize the recognition speed; the sixth part lists limitations of convolutional neural networks applied in rice disease identification and future research directions. Finally, a summary of the paper is presented.

## 2 Theory of convolutional neural network

The convolutional neural network is one of the classical ways of deep learning with multi-layers structure and convolution computation (Gu et al., 2018). It can automatically extract image properties and advanced features that cannot be extracted manually (Yamashita et al., 2018). It has obvious advantages in image recognition and can be applied to large-scale data training (Senan et al., 2020). The lightweight operation of a convolutional neural network is also a research hotspot recently (Haque et al., 2021).

### 2.1 Framework of convolutional neural network

**Figure 2:**
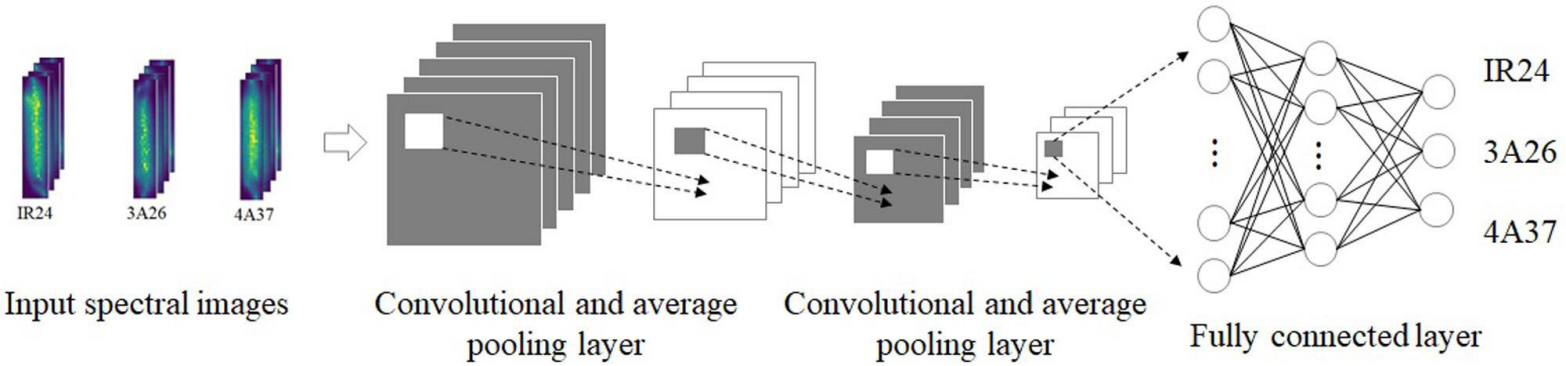
A convolutional neural network framework. (Reprinted from ref. (Zhang et al., 2020) under the terms of the Creative Commons CC-BY license).

The structure of a convolutional neural network can be split into the convolution layer, pooling layer, and fully connected layer according to different calculation ways (Zhang et al., 2020) (**Figure 2**). The feature map is generated by extracting local features of input data through the convolution layer. The feature map dimension is reduced by the pooling layer. The processed feature map is input into the fully connected layer and the outcome according to different tasks is output (Lu et al., 2017; N et al., 2021).

Convolution layer. The convolution layer collects local attributes of the training data by convolution operations (Atole and Park, 2018). The input data and weight parameters are combined with the offset value after convolution operations, and the result is the input of the activation function. The size of the final output feature graph is related to the setting of relevant parameters of the convolutional neural network. The formula for calculating convolution is:


$$x_{ij}^{\left(l\right)}=f\left(\sum_{m=0}^{k}\sum_{n=0}^{k}x_{i=m,j+n}^{\left(l-1\right)}w_{mn}^{\left(l\right)}+b^{\left(l\right)}\right)$$

x is the image pixel, k is the length or width of the convolution kernel, w is the weight vector, b is the bias, and f is the activation function. The image is input to the convolutional neural network as a data matrix, and the convolution kernel is essentially a local weight matrix as well. The convolution operation is to slide the convolution kernel over the input data in a certain number of steps, multiplying and summing element-by-element for each position to obtain a new 2D feature matrix. The convolution operation can effectively extract the local spatial information in the input data.

Pooling layer. Similar to the principle of convolution operations, pooling is realized by moving the sliding window on the feature graph (Jiang et al., 2020). According to the step size, each move on the feature map will get the presentation value of the region. After pooling operations, the size of the feature map will be reduced. The calculation formula for the pooling operation is the following:


$$x^{t}=R\left(\beta^{t}.P\left(x^{t-1}\right)+b^{t}\right)$$

R is the activation function, P is the pooling function, β is the weight coefficient, and b is the bias. The pooling operation divides the input feature mapping matrix into non-overlapping regions and then performs aggregation of each region by calculating the average or taking the maximum value. The pooling operation aggregates or counts the values within each window by performing a sliding window process on this matrix and outputs them to the next layer as input data. The pooling operation is usually immediately followed by the convolution operation, which reduces the feature dimensions of the output of the convolutional layer by pooling, effectively preserving the important feature information.

Fully connected layer. The fully connected layer is positioned in the last part of the convolutional neural network. The fully connected mode is adopted to map the two-dimensional feature map into a one-dimensional vector, and finally map to the sample space according to different tasks. The preceding convolution layer and pooling layer map the input data to the feature representation space, and then the fully connected layer maps the feature representation space to the sample’s label space to achieve the final classification or regression task (Islam et al., 2021).

### 2.2 Lightweight measures of convolutional neural network

Traditional convolutional neural networks have the problem of too many parameters. If the model is improved to obtain better classification performance, a lot of sample sets are needed. However, in practical engineering applications, it is hard to get a lot of sample sets to promote the classification precision. There is an increasing demand for convolutional neural network deployments on mobile terminals, and in-depth lightweight research on convolutional neural networks is required. Under the premise of not reducing model performance, architecture size and the computational amount should be reduced as far as possible to get a balance between performance and overhead (Hui et al., 2020). At present, the lightweight of convolutional neural networks is mainly realized through model compression and adjustment of the convolution operation.

The commonly model compression methods are network pruning, parameter quantization, low-rank approximation, and knowledge distillation.

**Figure 3:**
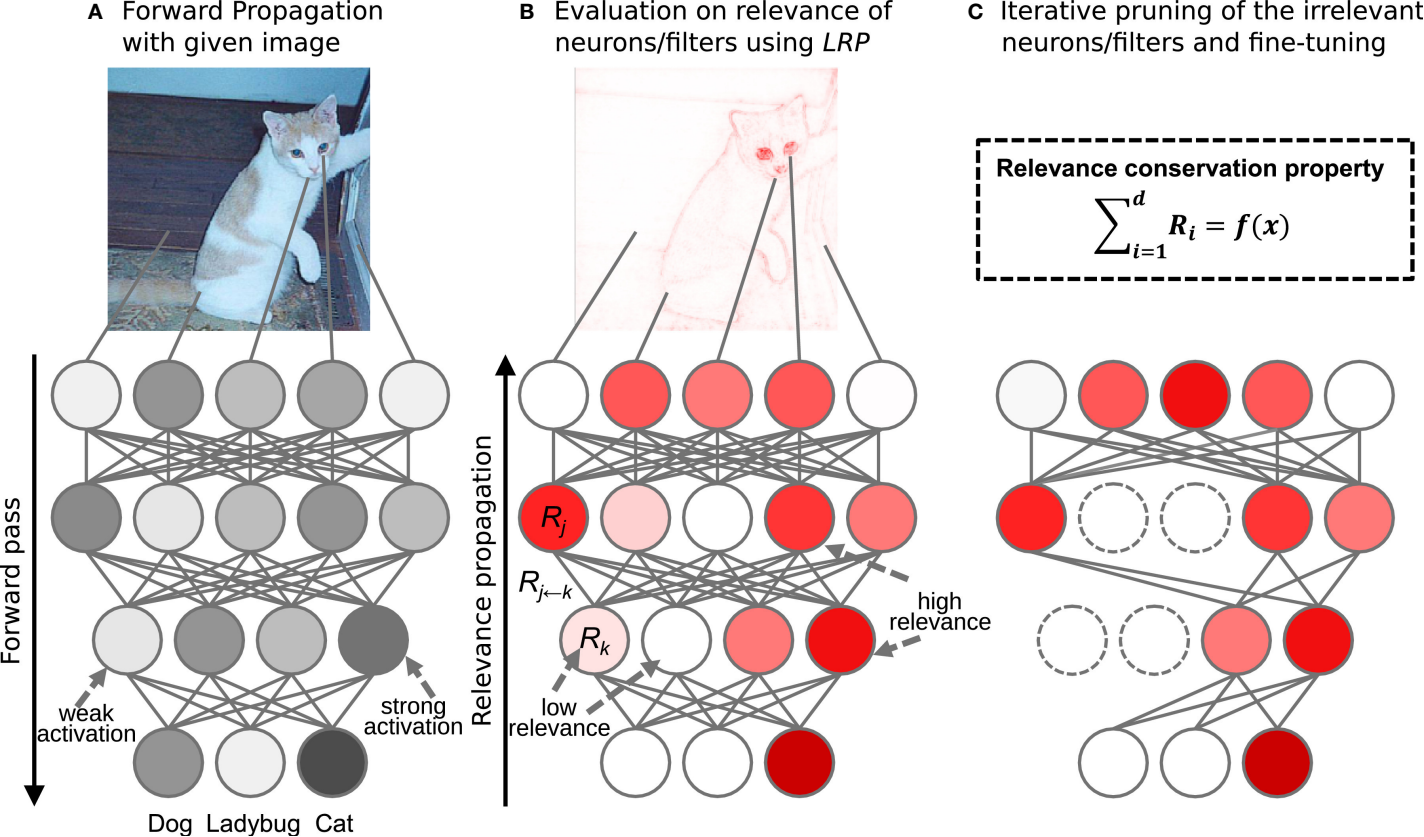
Schematic illustration of network pruning. **(A)** Original network; **(B)** Weight measure for each node; **(C)** Network after pruning. (Reprinted from ref. (Yeom et al., 2021) under the terms of the Creative Commons CC-BY license).

Network pruning. Pruning, as a classical technique in the field of model compression, has been widely used in the post-processing of various algorithms. Network pruning is an important technique which could reduce network complexity and prevent network overfitting (Yeom et al., 2021). It is widely employed in machine learning and convolutional neural networks (**Figure 3**). Network pruning can remove redundant connections in convolutional neural networks, decrease model complexity, and reduce the amount of computation. Moreover, it can effectually avoid overfitting and optimize the generalization of the architecture. Network pruning usually has three steps: training connection and measuring the importance of network neurons; removing unimportant neurons; retraining the network and fine-tuning the network (Chen R. et al., 2020).

**Figure 4:**
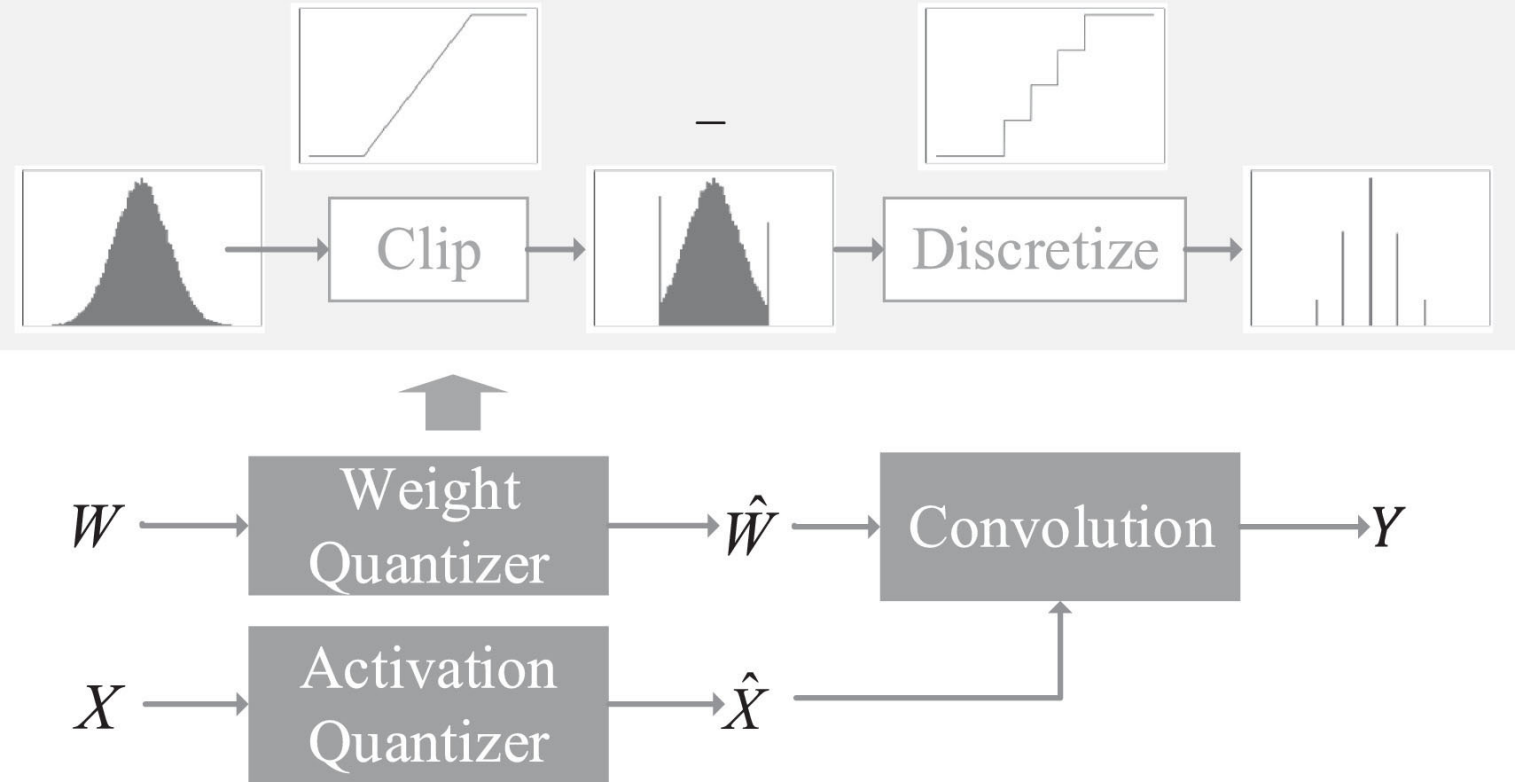
Quantization of a convolutional layer. (Reprinted with permission from ref. (Zhang and Chung, 2021) copyright 2021 Elsevier).

Parameter quantization. Quantization is operations of replacing all the original parameters with part parameters, which greatly reduces the storage overhead. Parameter quantization, as a common back-end compression technique, can obtain a large reduction in model volume but little performance loss (Zhang and Chung, 2021). The demerit is that the quantified network is fixed, making it hard to change (**Figure 4**). On the other hand, the generalization of this way is poor, resulting in high maintenance costs. One of the simplest quantization algorithms is scalar quantization (Zhang et al., 2022).

**Figure 5:**
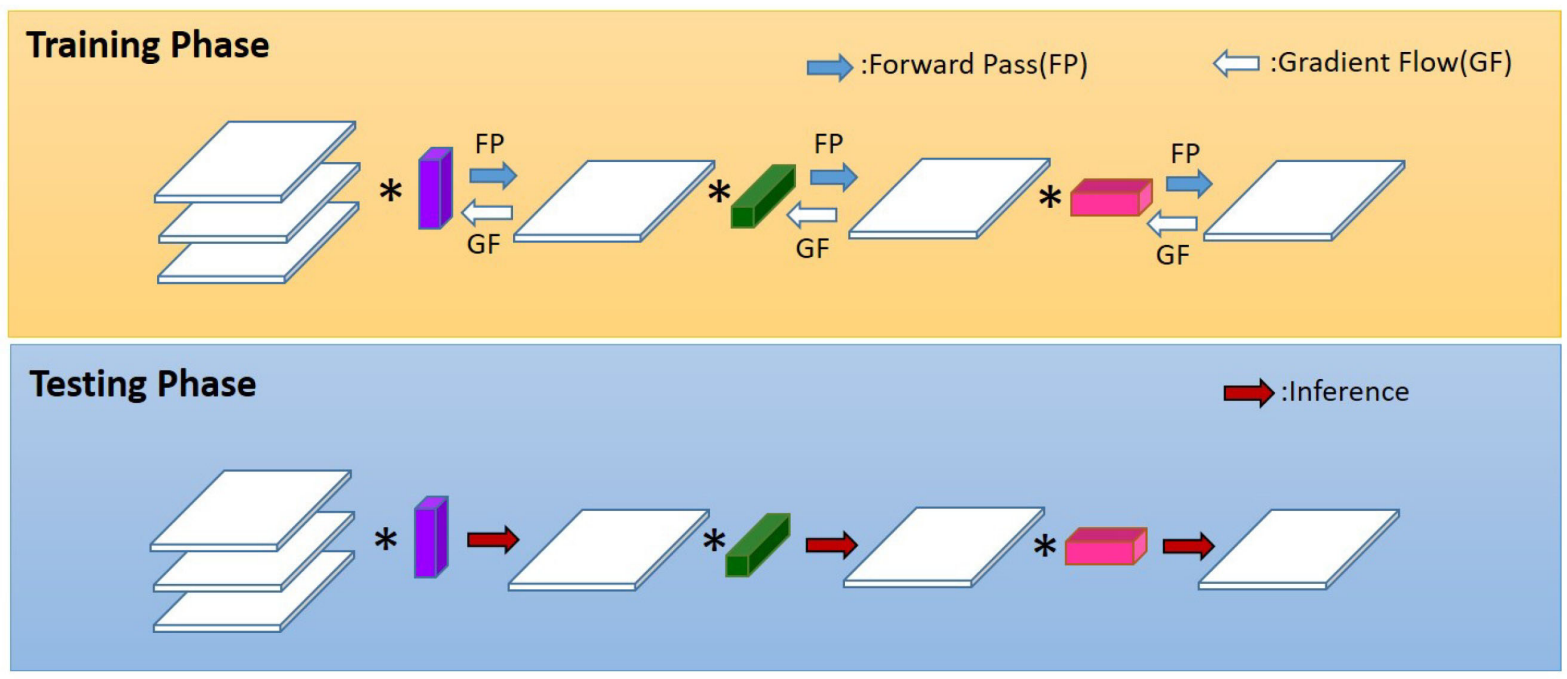
Rank-1 approximation. (Reprinted from ref. (Lee et al., 2021) under the terms of the Creative Commons CC-BY license).

Low-rank approximation. It decomposes the huge dense weight matrix into several small-scale matrices, the original weight matrix can be approximately reconstructed. The operation achieves the purpose of reducing storage and calculation (Lee et al., 2021). The basic computational mode of a convolutional neural network is convolution operations (**Figure 5**). In the actual implementation, the convolution operation is completed by matrix multiplication. However, the weight matrix tends to be dense and large in general, which brings huge overhead in computation and storage. An intuitive idea to solve the problem is that if the dense matrix can be approximately reconstructed through a few smaller matrices, then the storage and computation costs can be reduced effectively (Anvarjon and Kwon, 2020).

**Figure 6:**
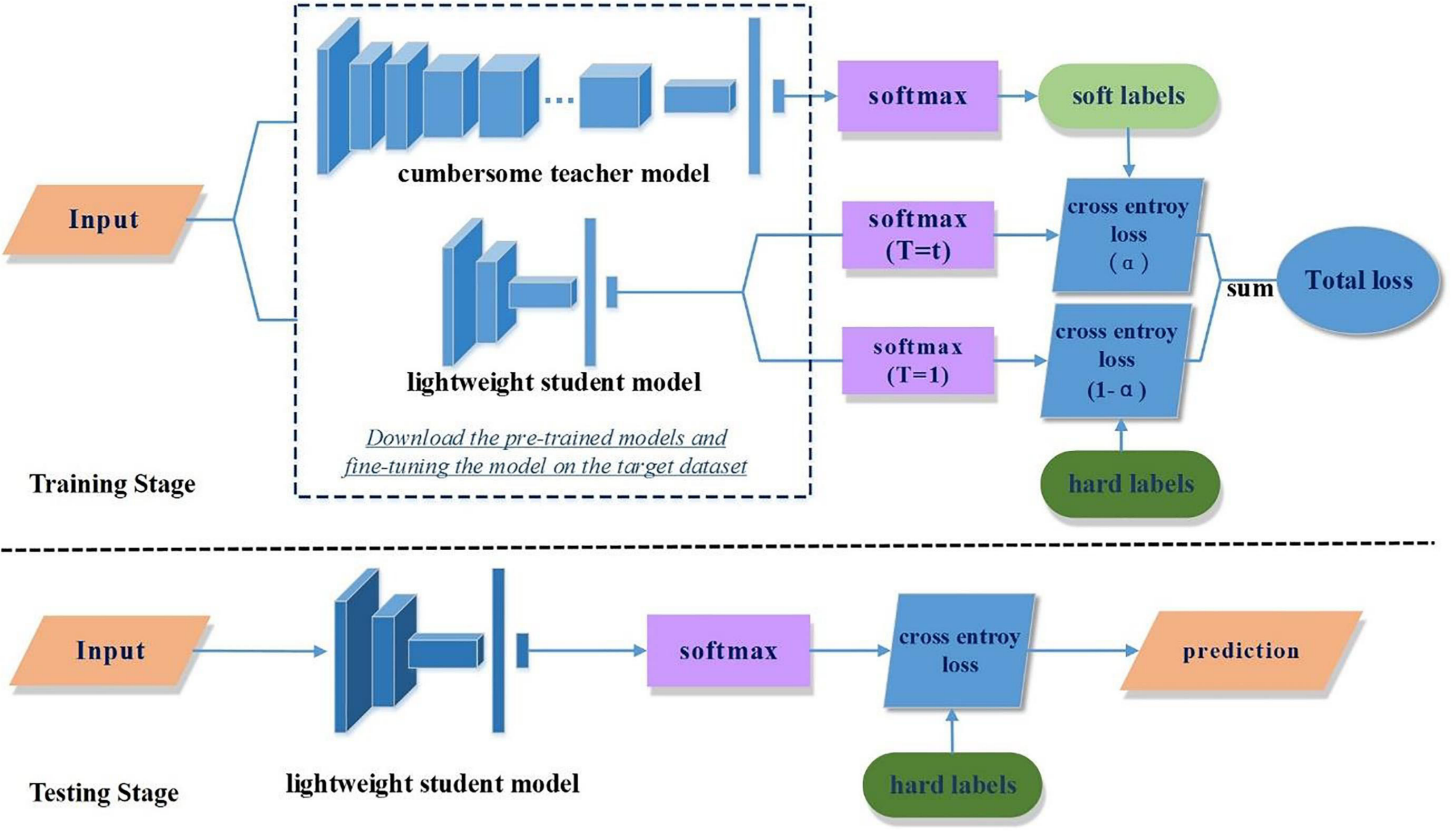
An example of knowledge distillation. (Reprinted with permission from ref. (Chen W. et al., 2022) copyright 2022 Elsevier).

Knowledge distillation. It is a transfer learning method, and its target is the convert of the knowledge learned from the complex network framework to the compact small model over certain approaches so that a tiny scheme can also obtain a similar capability as the complex model (Lee et al., 2021; Wang and Du, 2021; Chen W. et al., 2022) (**Figure 6**). In the framework of knowledge distillation, two basic elements play a decisive role: first, what is “knowledge”? That is, how to extract the knowledge in the model. The second is how to “distillation”? That is, how to complete the task of knowledge transfer (Sahu et al., 2018).

It is an effective method to adjust standard convolution, decrease convolution parameters and speed up convolution operation. Although the model compression takes a good part in the process of convolutional neural network lightweight, its process is too complex, and it usually requires detailed repeated training to achieve a similar performance to the original model. Therefore, some researchers begin to directly design lightweight convolutional neural network frameworks to control the number of parameters and computation by group convolution, depth-wise separable convolution, depth-wise convolution, and pointwise convolution (Sunija et al., 2021).

Group convolution. It divides the dimension of feature channels into several equal parts, then convolves them separately, and piles up the results. The idea of group convolution is widely used in network design. Besides reducing the number of parameters, it can also be regarded as a structured sparse approach, equivalent to a regularization manner. As the number of filter groups raises, the model parameters decrease, and the framework becomes more efficient. Since the convolution is partitioned into multiple paths and each path can be handled separately through a respective GPU, the architecture can be trained on several GPUs in parallel, and the training speed of the mode is greatly accelerated (Huang et al., 2019).

**Figure 7:**
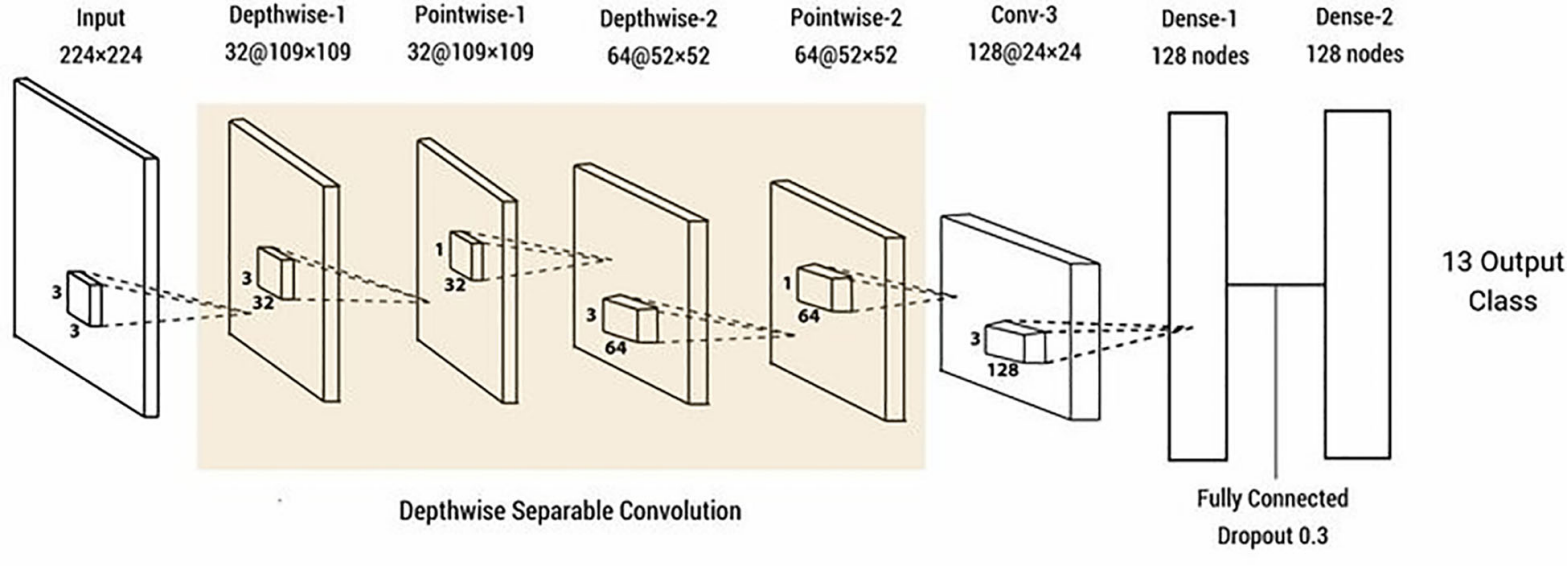
Schematic illustration of a convolutional neural network with depth-wise separable convolution. (Reprinted from ref. (Prottasha and Reza, 2022) under the terms of the Creative Commons CC-BY license).

Depth-wise separable convolution. The merit of depth-wise separable convolution is that the more attributes that need to be extracted, the more parameters can be saved, reducing the amount of computation. Deep-wise separable convolution is actually a kind of decomposable convolution operation, which involves spatial dimension, but also deals with depth dimension (Prottasha and Reza, 2022) (**Figure 7**). It can be decomposed into two smaller actions: deep convolution and point-by-point convolution.

Depth-wise convolution. It is a kind of packet convolution in which the number of packets is equal to the amount of feature channels, and the convolution kernel is one-to-one corresponding to the channels. Therefore, the depth of the output feature map is the same as that of the input. For the multi-channel characteristic graphs from the previous layer, all of them are first split into feature graphs of a single channel, and single-channel convolution is performed on them respectively and then stacked together. Different from the standard convolution activities, deep-wise convolution splits the convolution kernel into several channels and carries out convolution operations on each channel without changing the depth of the input property image. In this way, the output attribute graph with the same amount of channels as the input feature graph is acquired (Ullah et al., 2021).

Pointwise convolution. The deep-wise convolution only adjusts the dimension of the property graph from the previous layer, while the number of channels does not change, which demands to be modified by pointwise convolution. The pointwise convolution is a 1*1 convolution. Since deep-wise convolution does not merge information between channels, it needs to be applied together with pointwise convolution. It could raise or reduce dimension of feature graphs. The operation of pointwise convolution is similarly to the conventional convolution operation, which is to produce a new attribute map by weighted addition of the property map of the former stage in the depth direction. Each filter exports one feature map, so several channels require multiple filters (Gayathri et al., 2020).

## 3 Convolutional neural network for better recognition accuracy

Accuracy is the basis of all applications. Only with better recognition accuracy can convolutional neural networks be widely utilized in rice disease recognition. Small and scattered rice disease sites in the images can lead to poor identification accuracy. To solve this problem, many researchers have started to improve the models in terms of their feature extraction capability and training methods. To enhance the feature extraction capability of the model, multi-scale features of rice diseases are often extracted from the images and merged by introducing an attention mechanism. The improvement of the model training method is mainly implemented by using ensemble learning.

**Figure 8:**
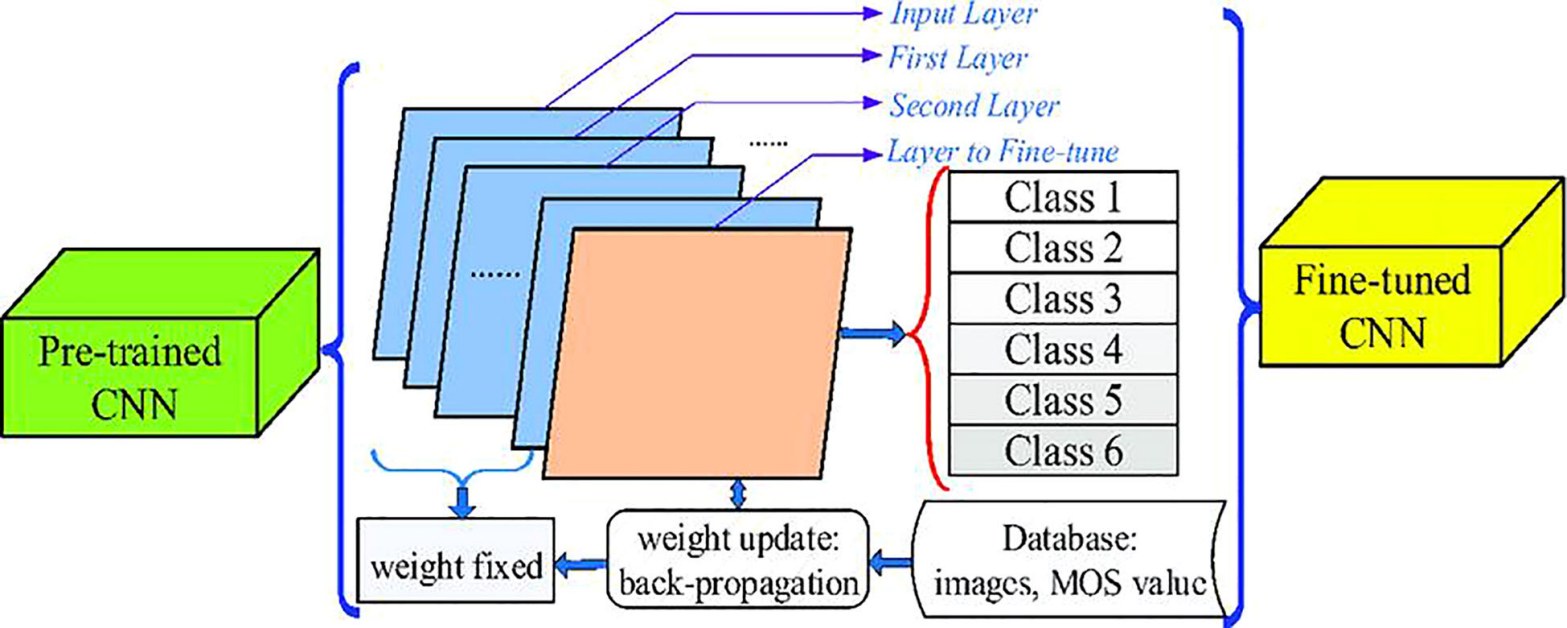
Fine-tuned CNN. (Reprinted from ref. (Bhujel and Shakya, 2022) under the terms of the Creative Commons CC-BY license).

By introducing the attention mechanism into a model, it can better understand the correlation between the input data, and improve its prediction ability. Shuai Feng et al. studied the detection of rice blast based on spectral characteristics (Feng S. et al., 2022). The convolutional neural network employed in the method combined the attention mechanism with a residual network to determine the optimal frontal characteristic wavelength. Guided gradient weighted class activation mapped spectral data to guided gradient weighted heat maps and finally determined the appropriate characteristic wavelength. Statistical analysis proved that this way could effectively identify the spectral characteristics of rice diseases and give a high precision recognition. The team also combined spectral features with vegetation index and texture features into the convolutional neural network to identify rice leaf blast (Feng S. et al., 2021). The scheme was adjusted based on a residual network. The spectral images of rice obtained by spectrometers in a paddy field in Shenyang, China, were increased from 145 to 4930 after data enhancement. The experimental comparison with several existing models verified that the neural network with multiple features was much better than the mode with only one feature. Shuo Chen et al. added an attention mechanism and multi-scale feature integration technique to the convolutional neural network to recognize rice diseases (Chen S. et al., 2021). The attention mechanism helped the model to find the key attributes, while the multi-scale feature integration technology could integrate the features of different scales for comprehensive analysis. The dataset consisted of 109 images taken by digital cameras in fields in Xuzhou, China and annotated by the LabelMe software. Data enhancement operations, such as rotation, stretching, sharpening, etc., led to a final dataset of 1,199 images. Experiments confirmed that the precision of rice disease recognition was improved. Mehdhar S. A. M. Al-Gaashani et al. introduced a self-attention mechanism to achieve high accurate rice disease recognition. The attention module embedded kernel attention and implemented contextual information extraction of features in this way. Experiments showed that the model had an accuracy of 98.71%. Afis Julianto et al. compared the accuracy of six typical convolutional neural networks: InceptionV3, ResNet50, InceptionResnetV2, DenseNet201, MobileNet, and EfficientNetB3 for rice disease recognition (Julianto and Sunyoto, 2021). Mendeley Data was used in the dataset, with 5,932 images covering four disease categories: white leaf blight, rice blast, brown spot, and tungro disease. The dataset was enhanced by zooming in, rotating, flipping, etc., and the image amount in the dataset was increased by six times. The experimental results reported that the InceptionResnetV2 network had the highest accuracy, but its training time was also the longest. Md. Mafiul Hasan Matin et al. modified the classical convolutional neural network AlexNet to identify rice diseases (Matin et al., 2020). Their proposed neural network had 5 convolutional layers, 3 fully connected layers, and finally 3 layers of customized network structure for rice disease recognition. The 120 images in the dataset came from Kaggle’s online data, and the number of images increased to 900 after data enhancement, involving leaf blight, brown spots, and smut of rice. The experiment verified that the accuracy of this method reached 99%. Wanjie Liang et al. also studied rice disease recognition based on the AlexNet network (Liang et al., 2019). They mainly investigated the automatic identification of rice blast disease. A total of 5,808 images were collected from the Institute of Plant Protection, Jiangsu Academy of Agricultural Sciences. The stochastic gradient descent algorithm was utilized to update the weights in the network during model training. The evaluation results revealed that the rice disease features extracted by this method had a better identification degree than those extracted by traditional manual methods, and the identification effect of rice disease was also better. Based on high-resolution images, Lele Wei et al. detected rice with different health states using the deep neural network YOLOv4 (Wei et al., 2022). All the images were taken by an unmanned aerial vehicle and processed by histogram equalization, color space conversion, and so on. The stochastic gradient algorithm based on momentum acceleration was employed to train the network mode. Compared with the conventional image segmentation algorithm, the results indicated that this technique was more efficient and stable. Susant Bhujel et al. combined cyclic learning rate fine-tuning and pre-training model in convolutional neural networks to recognize rice diseases (Bhujel and Shakya, 2022). The cyclic learning rate fine-tuning ensured a faster convergence, and the pre-training model could prevent the error signal amplification in the process of model training and speed up training (**Figure 8**). The original dataset contained 2,092 images from Kaggle’s online data, which was augmented to 8,368 images. Experimental data demonstrated that this method was effective in recognition.

Multiscale feature fusion can help a model locate the position of the target more accurately. By fusing features with different sensory fields, the model can find the boundary and detailed information of the target, thus improving the accuracy of target detection and localization. Ching-Ling Wang et al. conducted a series or parallel operation of multiple convolutional neural networks to find the best network structure for rice disease recognition (Wang et al., 2022). Each independent convolutional neural network extracted different features, and these features would be fused in the decision stage and output the final consequence. To further improve the recognition accuracy, they also carried out two-stage data enhancement on the training data. The experimental results indicated that the parallel multi-convolutional neural network structure had the highest recognition accuracy, but also could preserve the micro spot disease feature on rice leaves. He Liu et al. improved the accuracy of rice disease recognition by advance processing of training data and continuous tuning of hyperparameters of convolutional neural networks. All the images used for training the model were standardized to the same size and the dataset was augmented by data enhancement techniques. Parameters such as learning rate, batch size, and number of iterations were continuously adjusted during the model training process, and a framework with a recognition accuracy of 98.64 was finally obtained. T. Daniya et al. proposed a neural network architecture for high precision identification of bacterial leaf blight in rice. To ensure high recognition accuracy, the framework needed to carry out a range of actions on the input images (Daniya and Vigneshwari, 2022b). Firstly, noise suppression, pixel normalization, and segmentation would be carried out on the images. Then, feature extraction to the segmented images and selected effective recognition features was conducted. Finally, the identification result was output. Experimental results reported that this method had higher recognition accuracy than other mainstream ways. Narendra Pal Singh Rathore et al. researched a sequential convolutional neural network to detect diseases of rice and they obtained an accuracy of 99.1% (Rathore and Prasad, 2020). The training model utilized a dataset of 1,000 images, all from Kaggle. All images were compressed to remove redundant pixels. To speed up the training process, images were rotated, shifted, and clipped in the pre-processing stage. This model fully demonstrated the great potential of convolutional neural networks in rice disease recognition. N. V. Raja Reddy Goluguri et al. combined the convolutional neural network with support vector machine, artificial neural network, and long short-term neural network to test the recognition effect of the new model on rice disease (Goluguri et al., 2021). In the process of network parameter training, particle swarm optimization algorithm, artificial fish swarm algorithm, and efficient artificial fish swarm algorithm were utilized to optimize the weight of each neuron. The experimental data validated that the method optimized by the efficient artificial fish swarm algorithm combined with the convolutional neural network and the long short-term memory neural network had the highest accuracy in identifying rice diseases, reaching 97.5%. Ancy Stephen et al. performed high-precision rice disease identification by combining a generative adversarial network with a convolutional neural network. All training data was from Kaggle and uniformed as 224*224*3. The texture, color, and shape features in the target area were extracted in both grayscale and color values. The improved backtracking search algorithm then optimized the layers and nodes for generative adversarial neural network. The model achieved a final recognition accuracy of 98.7%.

**Figure 9:**
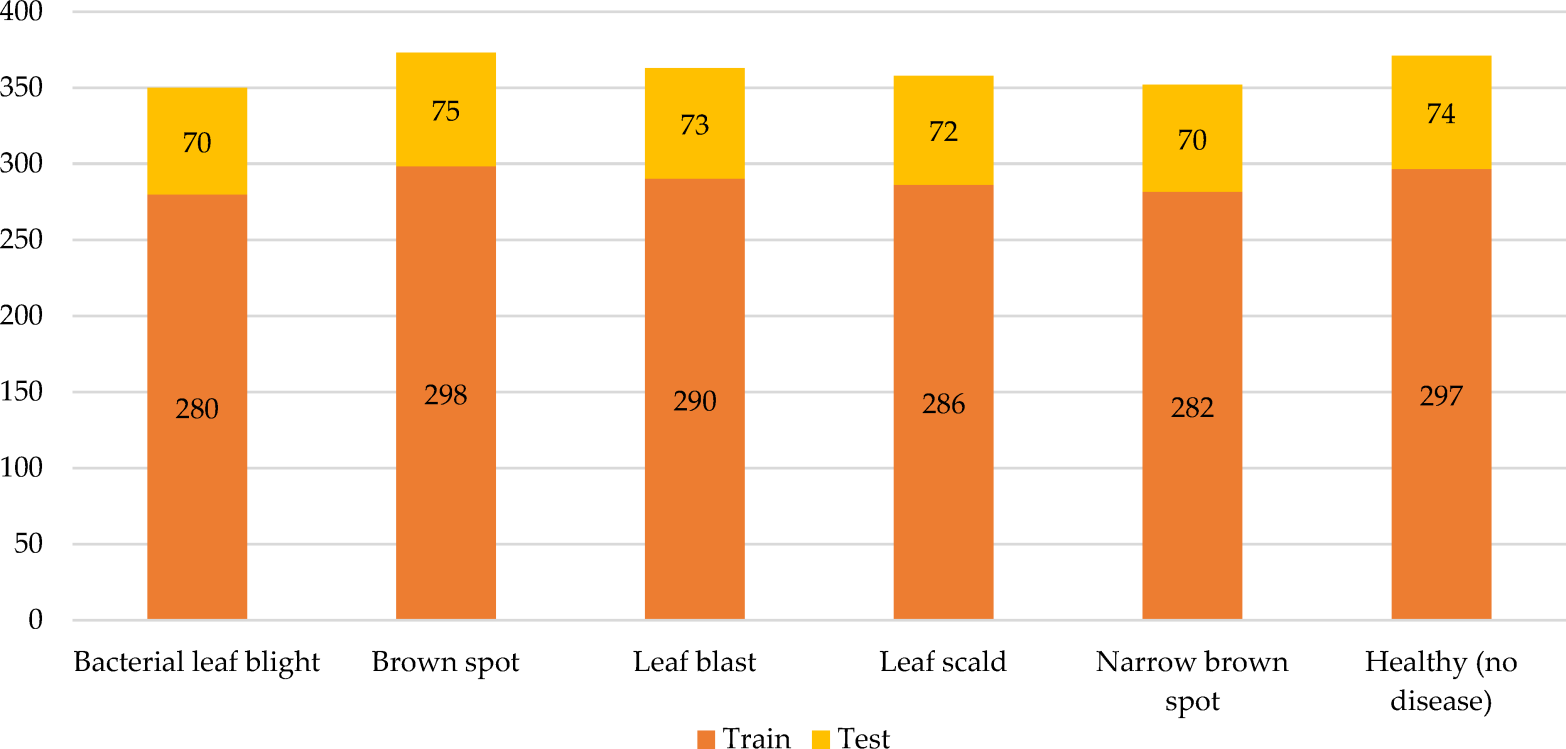
Distribution of training images and validation images of each rice disease and healthy rice leaf in the dataset. (Reprinted from ref. (Latif et al., 2022) under the terms of the Creative Commons CC-BY license).

Through transfer learning, a model can borrow features, relationships, or patterns from the source domain and apply them to the target task, thereby improving the model’s performance and generalization on the target task. Sudhesh K.M et al. applied transfer learning technology and dynamic pattern recognition decomposition method to identify rice diseases (K.M et al., 2023). Among multiple convolutional neural networks trained based on 3416 images, the DenseNet121 network had the best recognition effect on rice disease. Training other machine learning algorithms with the rice disease features extracted by the DenseNet121 network could often achieve better recognition results. In addition, dynamic pattern decomposition driven by an attention mechanism was added to the architecture to locate rice disease areas faster and more accurately. Experimental data showed that the recognition accuracy of this method reached 94.33%. Debaniranjan Mohapatra et al. applied the transfer learning technique to train the AlexNet network to recognize rice diseases (Mohapatra and Das, 2023). The dataset, which included 1,732 images from Kaggle’s online data, covered three rice diseases: rice leaf blight, brown spot, and smut. Since the AlexNet network had been trained in advance, the number of pictures in the rice disease dataset was much lower. Experiments data confirmed that the rice disease recognition accuracy of the method was up to 98.8%. Ghazanfar Latif et al. modified the convolutional neural network VGG-19 to identify rice diseases (Latif et al., 2022). The VGG-19 network had been trained on other datasets, and rice disease identification could be carried out just by fine-tuning parameters, which could not only accelerate the model training speed, but also ensure the effect. If there was too little data in the dataset, it was easy to overfit the framework. Therefore, the authors enhanced the images in the dataset through fine-tuning to add images, thus the effect of model training was ensured (**Figure 9**). In **Figure 9**, A rice disease dataset originally contained a small number of images, but it was expanded through data enhancement techniques such as rotation, scaling, and panning to bring the number of images within the dataset to 2,465. A portion of the data was used to train the convolutional neural network and other part of the data was used to validate the performance of the trained model. For example, there were 371 images of healthy rice, of which 297 images were used to train the network model while the remaining 74 images were used to validate the performance of the network model. The recognition results demonstrated that the method was more powerful than other existing ways.

## 4 Convolutional neural network for lightweight recognition

Rice disease recognition based on convolutional neural networks has obtained very good consequences in the laboratory, and the accuracy is particularly high. However, so far, the application in the process of rice cultivation is still relatively small. One of the most important reasons is that the convolutional neural network employed in the recognition process is still too complex, has too many parameters and high requirements on hardware. Therefore, further lightweight research on convolutional neural networks is needed to make it run on mobile devices such as smartphones.

It is very effective to process images through the deep neural network model to complete the classification and regression tasks. However, the parameters of the deep neural network model are generally tens of millions, even hundreds of millions. Such a complex model places high demands on both hardware and software computing resources during training and deployment. This is the reason that there are fewer examples of neural network technology application of rice disease identification. Currently, the lightweight operations of neural network models in rice disease identification process are achieved by adjusting the neural network structure.

Igor V. Arinichev et al. tested the performance of four relatively lightweight classical convolutional neural networks for rice disease identification: GoogleNet, ResNet-18, SqueezeNet-1.0, and DenseNet-121 (Arinichev et al., 2021). AlexNet and VGG networks were usually not considered in lightweight operations due to their complex structure and large number of parameters. The dataset contained 4,287 images related to brown spots and leaf blast in rice. The test results showed that the accuracy of these models all reached more than 95%. This indicated that convolutional neural networks played a very important role in rice disease recognition, and lightweight networks could also be deployed on mobile devices. Md. Sazzadul Islam Prottasha et al. proposed a relatively lightweight neural network with 2.4 million parameters to recognize rice diseases (Prottasha and Reza, 2022). To ensure the recognition effect under the premise of limited parameters, the Adam algorithm was applied to optimize and adjust parameters in the process of model training. The images in the dataset were from multiple rice fields in Bangladesh and the total number of images enhanced by the original data reached 13415. Experiments revealed that although the size of the model was smaller than the existing 8 most advanced convolutional neural networks, it still had a good recognition effect.

Yibin Wang et al. added the attention mechanism and Bayesian optimization algorithm to a convolutional neural network to reduce its volume and finally realized the application of rice disease recognition on mobile devices (Wang et al., 2021). The attention mechanism allowed the convolutional neural network to understand long distance information within the mode. Bayesian optimization applied a posterior function to decrease the parameters of the convolutional neural network. The dataset contained 2370 pieces of data, including both diseased and healthy rice samples. The extracted features were evaluated visually based on activation mapping and filter visualization techniques. Cross-validation classification experiments showed that the precision of the architecture achieved 94.85%. Chowdhury R. Rahman et al. presented a two-stage mini-convolutional neural network for offline recognition of rice diseases (Rahman et al., 2020). Compared with hundreds of millions of parameters in traditional convolutional neural networks, the network only needed 800,000 parameters, which made the memory efficiency very high and suitable for application in mobile scenarios. A total of 1426 images were included in the dataset for the training of the framework, involving five rice disease categories: pseudosmut, smut, sheath blight, health, and others. All the images were taken with four different types of cameras in different situations, such as summer and winter. Experiments indicated that the model achieved a good balance between memory efficiency and recognition accuracy. Junde Chen et al. designed a convolutional neural network called MobInc-Net for rice disease recognition (Chen J. et al., 2022). To reduce the complexity of the model, the original convolution methods were replaced by deep convolution and point convolution. A total of 1,000 images were collected into the dataset, covering 12 categories, through live photography and crawling on the web. The two-stage transfer learning technique used in mode training further developed the function of the method. Experimental data verified that the procedure had satisfactory efficiency and accuracy even in the complex background of rice disease recognition. The team also studied another outstanding lightweight convolutional neural network to identify minor lesions of rice diseases (Chen J.et al., 2021). This scheme belonged to the category of transfer learning. Training was started on a convolutional neural network that had been trained on ImageNet, which not only reduced the training cost but also improved the training effect. In addition, the framework also introduced an attention module, which could extract more critical details during feature extraction. The dataset consisted of 1,100 images, 660 of which were got online and other 440 were taken in paddy fields. Experiments based on both public and local datasets demonstrated the validity and reliability of the proposed method.

**Figure 10:**
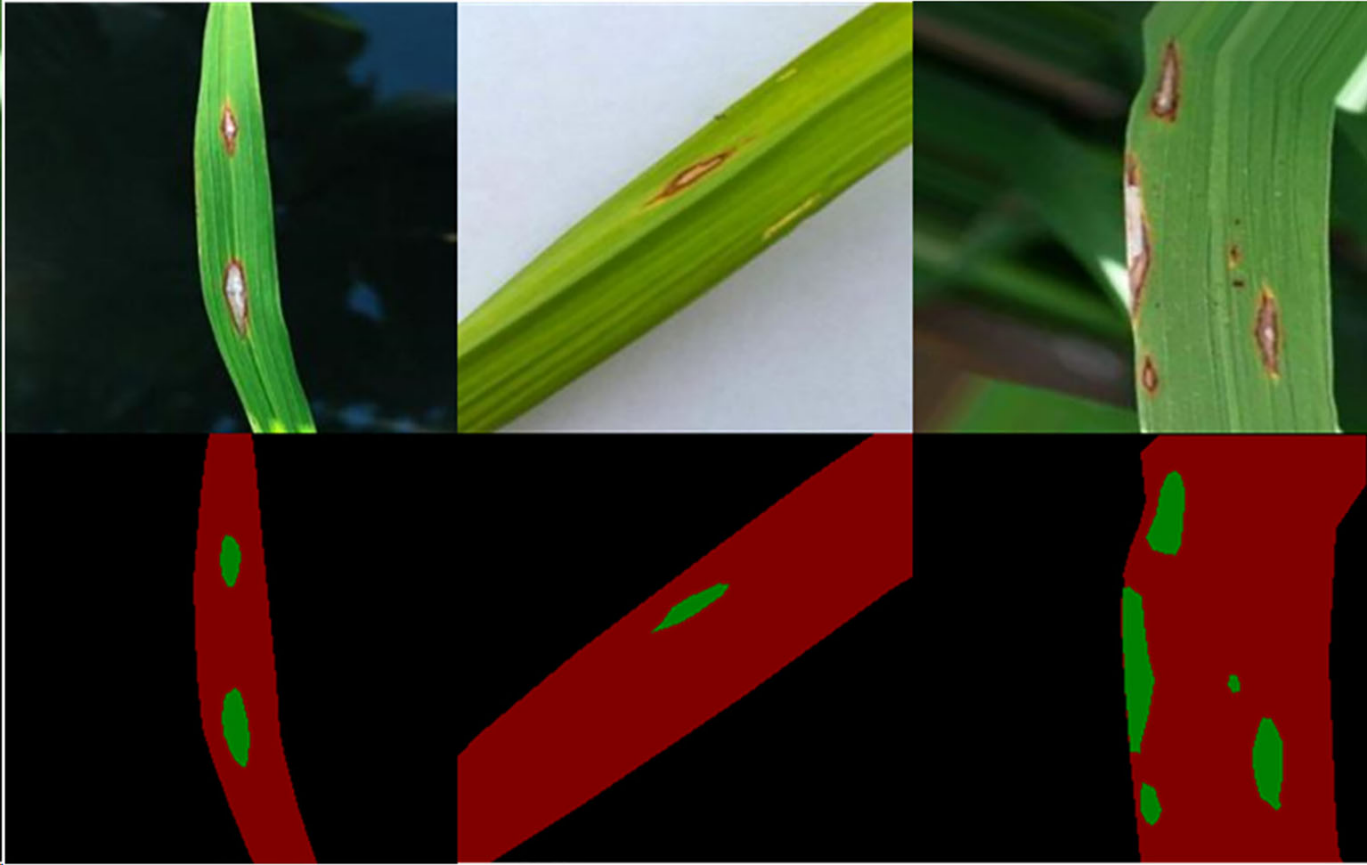
Original images and labeled images: the black part means background, the red part means healthy rice leaf, while the green part means rice disease spot. (Reprinted from ref. (Feng C. et al., 2022) under the terms of the Creative Commons CC-BY license).

Poornima Singh Thakur et al. investigated a lightweight convolutional neural network with 7 convolution layers for rice disease recognition named VGG-ICNN (Thakur et al., 2023). The network had about 6 million parameters, much less than traditional convolutional neural networks, but its multi-scale feature extraction ability was still very good. The mode had been tested on several public datasets such as PlantVillage, and the results validated that it had a very good recognition effect. The network not only had a good recognition effect on rice diseases but also had perfect performance on maize, apple, and other plant diseases. The performance was also as well as other lightweight convolutional neural networks such as EfficientNet B0. Changguang Feng et al. incorporated deep feature extraction and the attention mechanism into a convolutional neural network to recognize rice blast disease (Feng C. et al., 2022). The depth feature extraction module could simultaneously extract the deep and shallow features of rice blast, and the attention mechanism module could achieve the lightweight goal of this neural network. The dataset contained 800 images, selected from the open dataset, and annotated with the LabelMe software (**Figure 10**). **Figure 10** showed an image which contained rice disease labeled by the software LableMe. The background in the labeled images was standardized to black, the rice leaves were standardized to red, and the portion that was standardized to green represented the location of the lesion. The experiment showed that the method had good universality ability, strong anti-interference ability, and achieved a good balance between recognition speed and accuracy. At the same time, the method supplied a good reference for the deployment of rice disease recognition programs on the Internet of Things and mobile devices.

**Figure 11:**
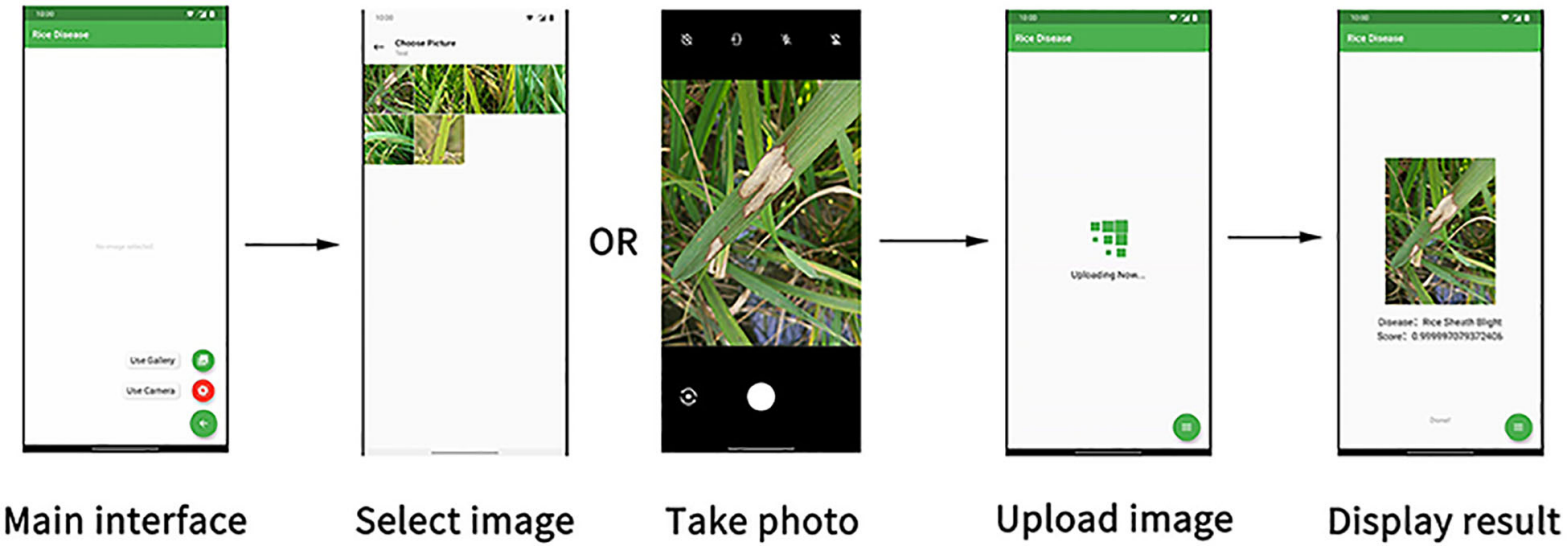
The interface of the rice disease recognition APP. (Reprinted from ref. (Deng et al., 2021) under the terms of the Creative Commons CC-BY license).

Ruoling Deng et al. developed a deep learning program based on a convolutional neural network that could run on a smartphone and identify rice diseases (**Figure 11**). The method integrated several sub-modules to distinguish different rice diseases as much as possible (Deng et al., 2021). The training dataset had 33,026 images covering six rice diseases: leaf blast, pseudosmut, neck blast, sheath blight, bacterial stripe, and brown spot. Instead of starting from scratch, a mature scheme already trained on ImageNet was taken to the dataset for training, greatly reducing the training time. The test verified that the recognition accuracy of the model could still reach 95%. Dengshan Li et al. used deep learning techniques to examine rice pests and diseases in the videos (Li et al., 2020). The video was first divided into frames, which conducted rice diseases detectionand then resynthesized into video. The dataset contained 5,320 photos taken by mobile phones in rice paddies in several Chinese provinces, which related to rice grain blight, borers, and brown spots. In the experiment, the framework had achieved satisfactory results and the recognition results of the unprocessed original video were also very good, even though the video was fuzzy and the recognized object was irregular. Jiapeng Cui et al. presented an optimized lightweight convolutional neural network to identify rice diseases, which could identify not only the species of rice diseases, the location of rice diseases (Cui and Tan, 2023). They transposed convolution upsampling in the neural network structure and expanded convolution downsampling to achieve better extraction of rice disease features. The team utilized a digital camera to capture 500 images about rice leaf blight, stripe blight, white spot, stripe blight, and leaf blast. 2,500 images formed the dataset for training the neural network. The results revealed that the method was effective in identifying the five rice diseases.

## 5 Convolutional neural network for higher recognition speed

It is greatly significant to improve the speed of rice disease recognition and realize real-time applications. A lot of researchers have done much work on this aspect and have achieved good results. Rice disease identification based on specific types of images and the use of specific algorithms in the identification process can improve the speed of rice disease identification.

**Figure 12:**
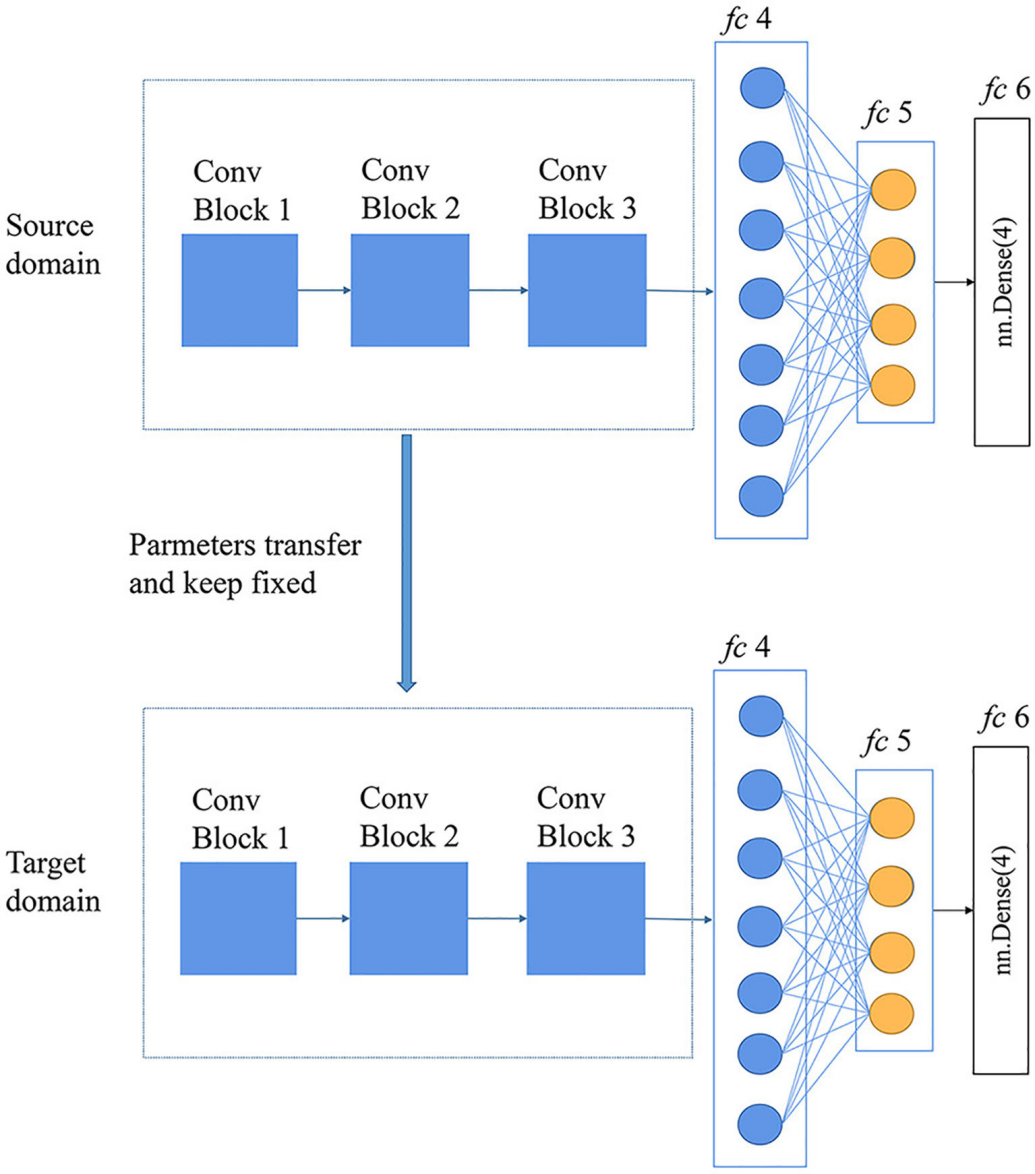
Schematic diagram of the fine-tuning algorithm. (Reprinted from ref. (Feng L. et al., 2021) under the terms of the Creative Commons CC-BY license).

Many studies have demonstrated that identification based on rice disease images obtained by hyperspectral techniques and terahertz imaging is often faster. Lei Feng et al. introduced hyperspectral imaging technology and transfer learning into the process of rice disease detection and realized rapid recognition (Feng L. et al., 2021). Hyperspectral imaging was used to obtain images of rice diseases (**Figure 12**). The convolutional neural network trained on the original dataset was adjusted and utilized for rice disease recognition. Methods of adjustment included fine-tuning, depth-dependent alignment, and depth-domain obfuscation. The results showed that the model had high detection efficiency and generalization performance, and the scheme could detect different kinds of rice disease. Jinnuo Zhang et al. studied white leaf blight at the stage of rice breeding, using terahertz imaging and near-infrared spectral imaging (Zhang et al., 2020). The recognition models of one-dimensional spectral image and two-dimensional spectral image were respectively constructed with a convolutional neural network. One-dimensional spectral images contained spectral information of samples, while two-dimensional spectral images included both spectral information and spatial information of samples. To better investigate the recognition process, the team also performed a visual analysis. They finally confirmed that the combination of terahertz absorption spectra and convolutional neural network could perform the detection quickly.

Of course, the time required for identification can also be reduced if the common rice disease images are properly processed. Santosh Kumar Upadhyay et al. devised a detection architecture with a convolutional neural network to obtain rapid diagnosis of rice diseases (Upadhyay and Kumar, 2022). From the input image to output result, the model contained 22 layers. Three rice diseases could be identified by the method: leaf smut, black spot, and leaf blight. Each disease had 4000 pictures in the dataset, of which 3200 pieces were the training set and 800 pieces were the verification set. All images were adjusted to a size of 64 by 64, then the background noise was removed and then grayed, and binarized. The advantage of the operations was that fast identification was guaranteed. The results revealed that the method was fast and effective, and the accuracy gained 99.7%.

Researchers also found that the recognition model based on the Faster R-CNN architecture was better than other algorithmic models in terms of rice disease recognition speed. An area based convolutional neural network was proposed by Bifta Sama Bari et al. to achieve real-time recognition of rice disease (Bari et al., 2021). The enhancement of the regional proposal network improved the detection speed of rice disease greatly. The dataset included both public data on the Internet and photos taken by the authors in the field, and there were 2400 pictures in the dataset. Augmentations to the images in the dataset further promoted the recognition speed and efficiency of the architecture. The results showed that the framework could realize real-time recognition of common rice diseases with very high accuracy. In addition, it could recognize healthy rice leaves. Prabira Kumar Sethy et al. reported a mode to recognize false smut of rice which combined a region proposal network and a convolutional neural network (Sethy et al., 2020b). The region proposal network could assist the model quickly locating the area of disease in the image to speed up the identification activities. All the images in the dataset were obtained by the camera on the smartphone and annotated in the MATLAB software. The test confirmed that the algorithm was effective. But the test may be invalid in some cases. The reason for invalid detection may be that the model provided multiple proposal regions, therefore the system performance needed to be further optimized. Taha Hussain et al. designed a rapid rice disease detection framework based on a convolutional neural network (Hussain et al., 2021). To improve the detection speed, the maximum pooling strategy was adopted in the pooling layer. The 2000 training images and 589 verification images in the dataset were captured by SLRS. All the images were processed into regular patterns and the color images were converted to gray level to ensure the speed of training and recognition. The experimental consequences demonstrated that the framework could complete the recognition procedure in only 0.05 seconds when the recognition accuracy reached 95%, which was very fast.

## 6 Limitations and prospect

Although convolutional neural networks have achieved a lot of results in rice disease recognition, there are still some problems that restrict the development of this research. It is expected that the key work of the research in the future will focus on solving these difficulties.

### 6.1 Limitations of convolutional neural network in rice disease recognition

Although rice disease recognition based on convolutional neural networks has achieved good results, it still faces the problems of excessive training data, limited accuracy, and high computer computation consumption in practical applications (Joseph et al., 2023).

Rice disease recognition based on convolutional neural networks and images requires a large amount of data to train the model and validate its performance. Different types of rice diseases may behave similarly on images, and a large number of images must be available to ensure the effectiveness of recognizing different diseases (Ahmed et al., 2023). The convolutional neural network, as a deep learning model, has a complex structure and contains many parameters, so the demand for data during training is relatively large (Aggarwal et al., 2023; Dogra et al., 2023). Therefore, the rice disease recognition work based on convolutional neural network must have enough rice disease images to ensure the final recognition effect. With the increasing requirement of rice disease recognition accuracy in real-world applications, it becomes more and more difficult to accomplish the work using convolutional neural networks alone. Many researchers have worked on optimizing the identification of rice diseases by modifying the structure of convolutional neural networks. Initially, they achieved good results, but as the optimization work progressed, the improvement in the accuracy of rice disease recognition became less effective (Lamba et al., 2023). To train and obtain a convolutional neural network that is effective in recognizing rice diseases, massive computational resources are often required (Haridasan et al., 2022; Bhuyan et al., 2023). The method relays significantly on the hardware platform, thus greatly limiting the real-time and mobile applications of convolutional neural networks in the rice disease identification process (Patil and Kumar, 2022; Senthil Pandi et al., 2022). Convolutional neural network-based rice disease recognition has to solve this problem if it aims to get out of the laboratory and gain wide application.

### 6.2 Prospect of convolutional neural network in rice disease recognition

Rice disease recognition based on convolutional neural networks still has vast research space and application value in the future. First, rice disease identification based on small sample data may be a hot spot in the future research. Enhancing the model’s ability of extracting rice disease features by modifying the structure of convolutional neural networks can improve the training speed and extend the application range. Expanding the small sample dataset by data augmentation techniques or employing transfer learning methods to enhance the extraction ability of specific features by convolutional neural networks are the two feasible approaches (Lu et al., 2023; Ramkumar Raja et al., 2023). Second, coupling convolutional neural networks with other machine learning models through an ensemble learning approach may further enhance the recognition of rice diseases (Ahad et al., 2023; Yang et al., 2023b). Different types of machine learning models are not sensitive to the features in the dataset, and integrating multiple machine learning models together can provide more comprehensive and effective recognition of various features. Therefore, it will improve the accuracy of recognition. Third, real-time identification and mobile deployment of rice diseases will also be the focus of the following research. Only when real-time identification of rice diseases is realized on mobile devices such as smartphones can a wide range of applications be truly realized. Currently, there are still many factors constraining the real-time rice disease identification and mobile deployment, such as complex network structure and large number of training data. Therefore, this research will be the focus of the next long period of time (Yang et al., 2023a).

## 7 Summary

Rice is one of basic foods in the world, and it is greatly significant to ensure its growth and yield security. From growth to harvest, rice is threatened by a variety of diseases, which poses a serious challenge to human food security. To prevent and control rice diseases, human beings began to study and control rice diseases from a very early time. Traditionally, farmers judge rice disease types based on the existing experience, which takes a lot of trouble and effort, and there are serious defects in recognition accuracy and large-scale promotion. As a very successful branch of deep learning technology, the convolutional neural network has made remarkable achievements in image-based classification, recognition, and segmentation. Automatic recognition of rice disease based on convolutional neural networks has also gained many effects in recent years. This paper summarizes the excellent achievements of convolutional neural networks in rice disease recognition in recent years and focuses on its research progress in recognition accuracy improvement, recognition speed optimization, and mobile terminal deployment. The current research on rice disease recognition based on convolutional neural networks mainly focuses on the improvement of accuracy, and the recognition accuracy in the laboratory has reached a high level through data preprocessing and network structure optimization. To extend the application of convolutional neural networks for rice disease recognition in practice, it is necessary to improve its recognition speed and the ability of deploying in mobile devices. Therefore, the simplification of the network structure is also the focus of the research. As convolutional neural networks require a large amount of sample data to support the training process, the needs for computer computation power are also relatively high, which limits the application of convolutional neural networks in rice disease identification. In the future, the convolutional neural network training based on small sample data combined with transfer learning technology, performance optimization of convolutional neural networks combined with ensemble learning technology, and simplification of the network structure for mobile deployment and real-time detection are the focus of convolutional neural networks in rice disease recognition. It is believed that this paper is important for further boosting the capability of convolutional neural networks in rice disease recognition and expanding the utilization of convolutional neural network in rice disease recognition.

## Author contributions

HN: Writing – original draft, Writing – review & editing. SL: Writing – original draft, Writing – review & editing. QZ: Writing – review & editing. TZ: Conceptualization, Writing – review & editing.
