# Supplementary material for: Convolutional neural network in rice disease recognition: accuracy, speed and lightweight
Source: Front Plant Sci. 2023 Nov 1;14:1269371. doi: 10.3389/fpls.2023.1269371 (PMC10646333; doi:10.3389/fpls.2023.1269371)
Supplement: Supplementary file 1 [file DataSheet_1.docx]

Supplementary Material

# Supplementary Data

Not applicable.

# Supplementary Figures and Tables

## Supplementary Figures


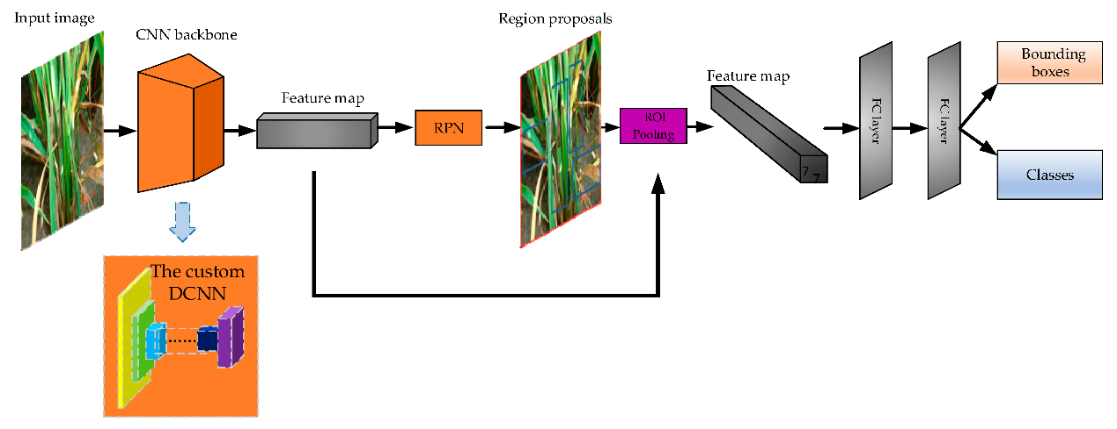


**Supplementary Figure 1**. Rice disease detection model diagram. (Reprinted from ref. [Li et al., 2020] under the terms of the Creative Commons CC-BY license.)


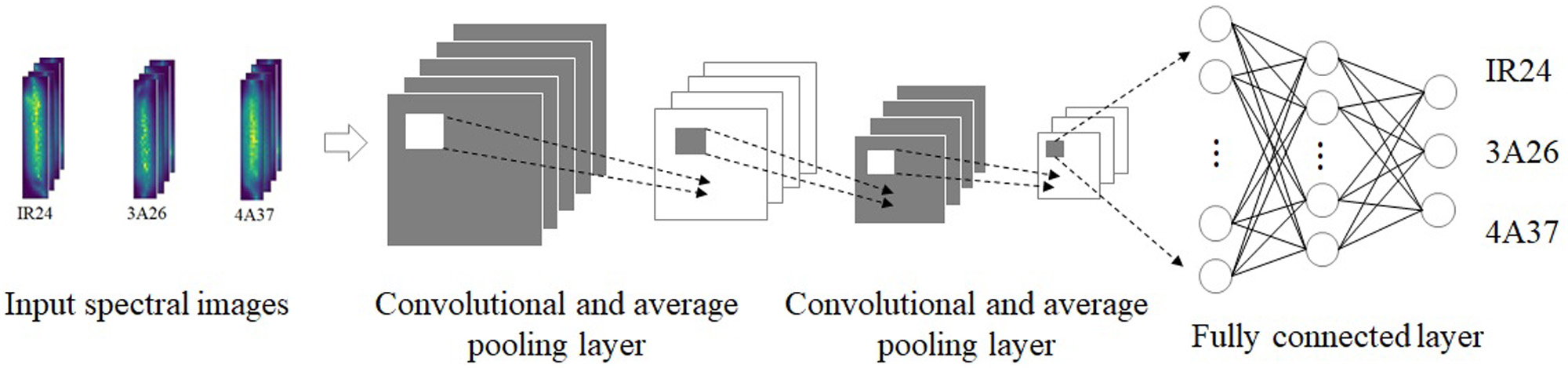


**Supplementary Figure 2.** A convolutional neural network framework. (Reprinted from ref. [Zhang et al., 2020] under the terms of the Creative Commons CC-BY license.)


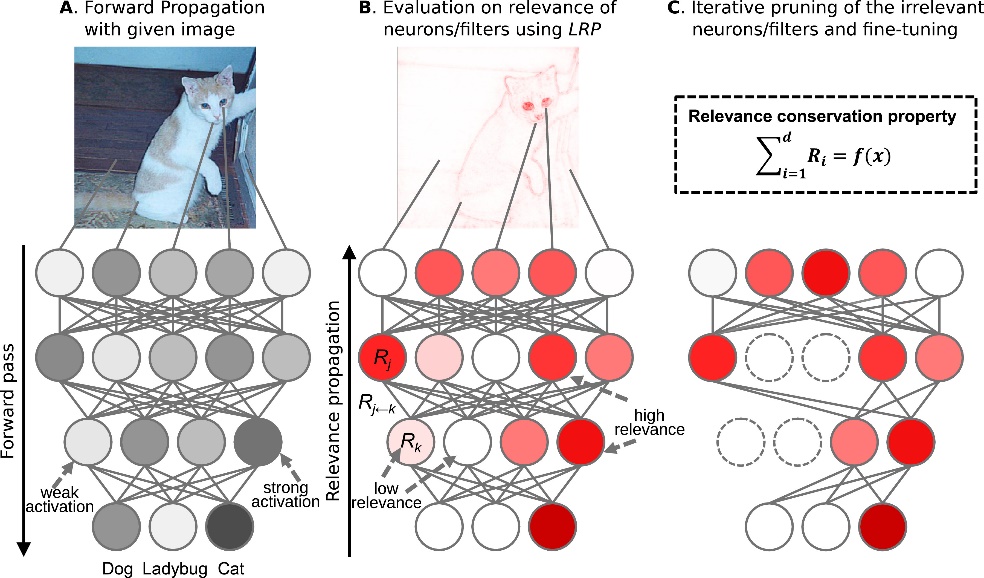


**Supplementary Figure 3.** Schematic illustration of network pruning. (A) Original network; (B) Weight measure for each node; (C) Network after pruning. (Reprinted from ref. [Yeom et al., 2021] under the terms of the Creative Commons CC-BY license.)


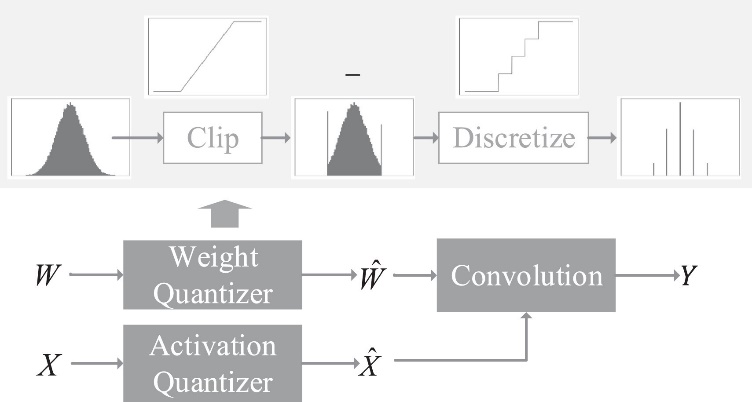


**Supplementary Figure 4.** Quantization of a convolutional layer. (Reprinted with permission from ref. [Zhang and Chung, 2021] copyright 2021 Elsevier.)


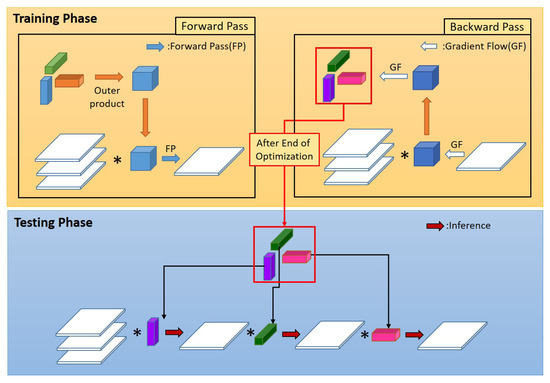


**Supplementary Figure 5.** Rank-1 approximation. (Reprinted from ref. [Lee et al., 2021] under the terms of the Creative Commons CC-BY license.)


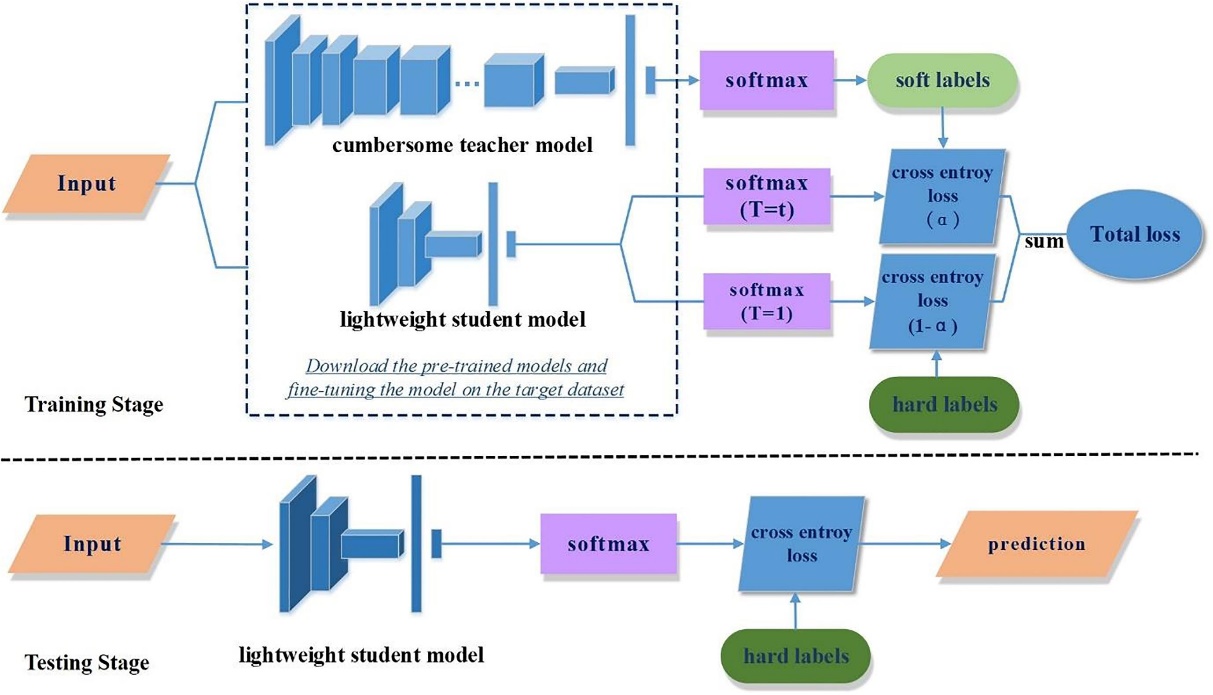


**Supplementary Figure 6.** An example of knowledge distillation. (Reprinted with permission from ref. [Chen et al., 2022b] copyright 2022 Elsevier.)


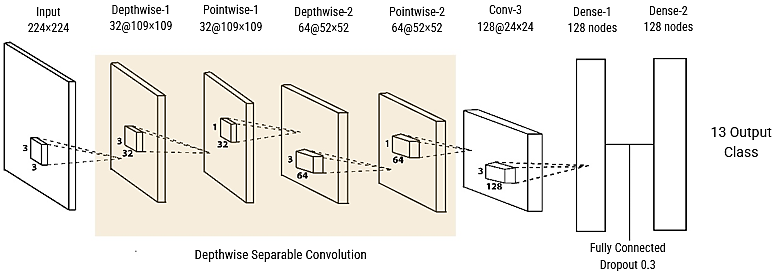


**Supplementary Figure 7**. Schematic illustration of a convolutional neural network with depth-wise separable convolution. (Reprinted from ref. [Prottasha and Reza, 2022] under the terms of the Creative Commons CC-BY license.)


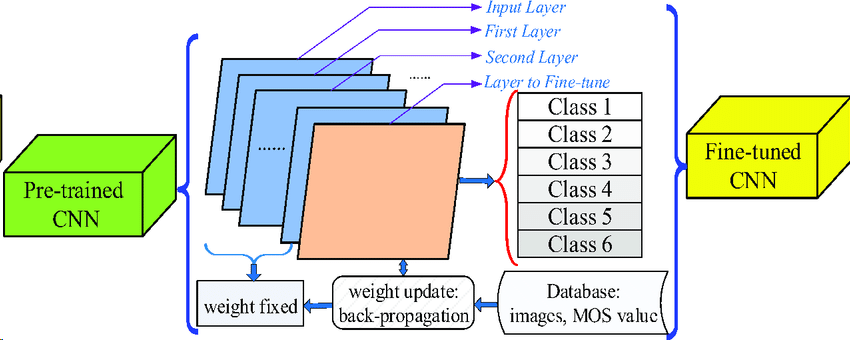


**Supplementary Figure 8.** Fine-tuned CNN. (Reprinted from ref. [Bhujel and Shakya, 2022] under the terms of the Creative Commons CC-BY license.)


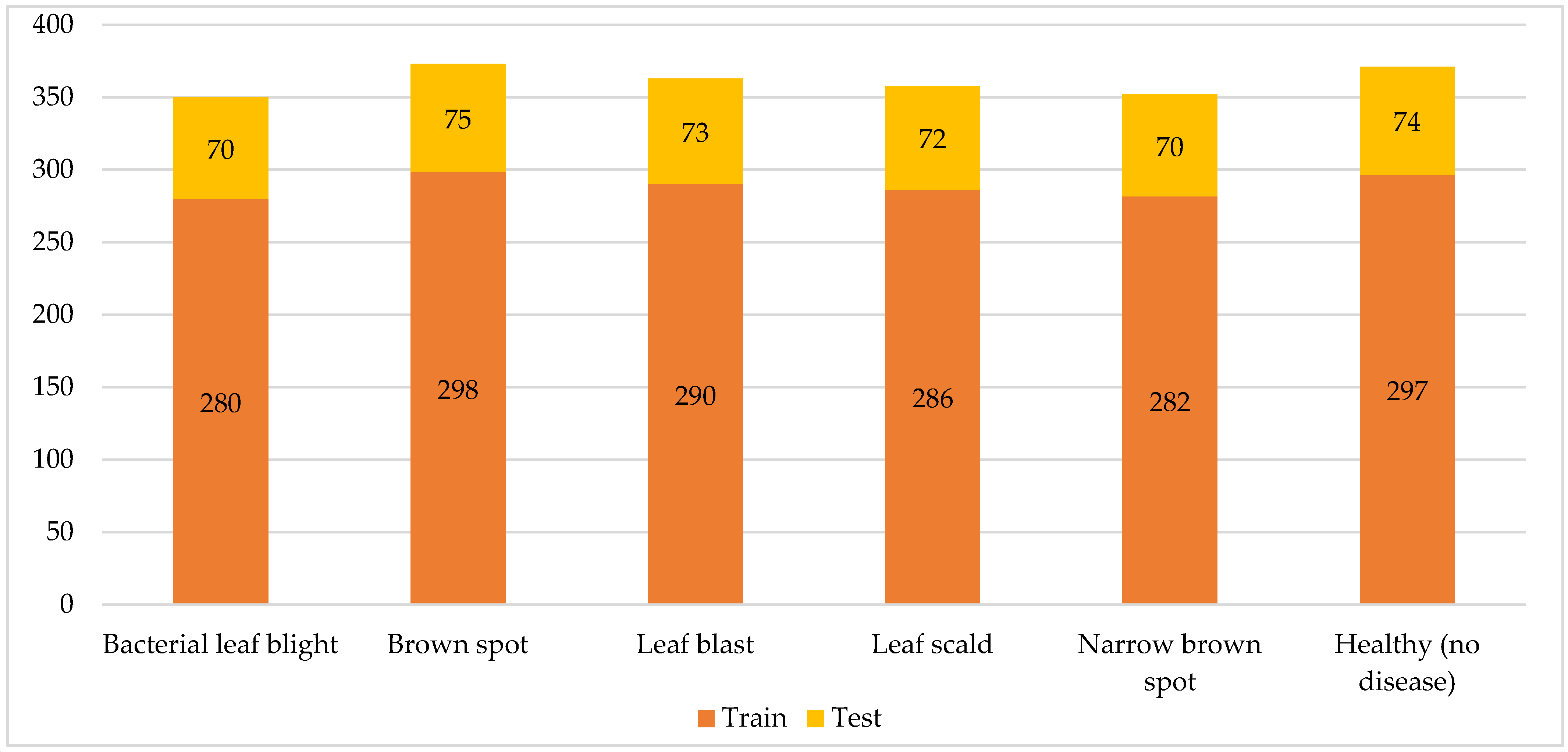


**Supplementary Figure 9.** Distribution of training images and validation images of each rice disease and healthy rice leaf in the dataset. (Reprinted from ref. [Latif et al., 2022] under the terms of the Creative Commons CC-BY license.)


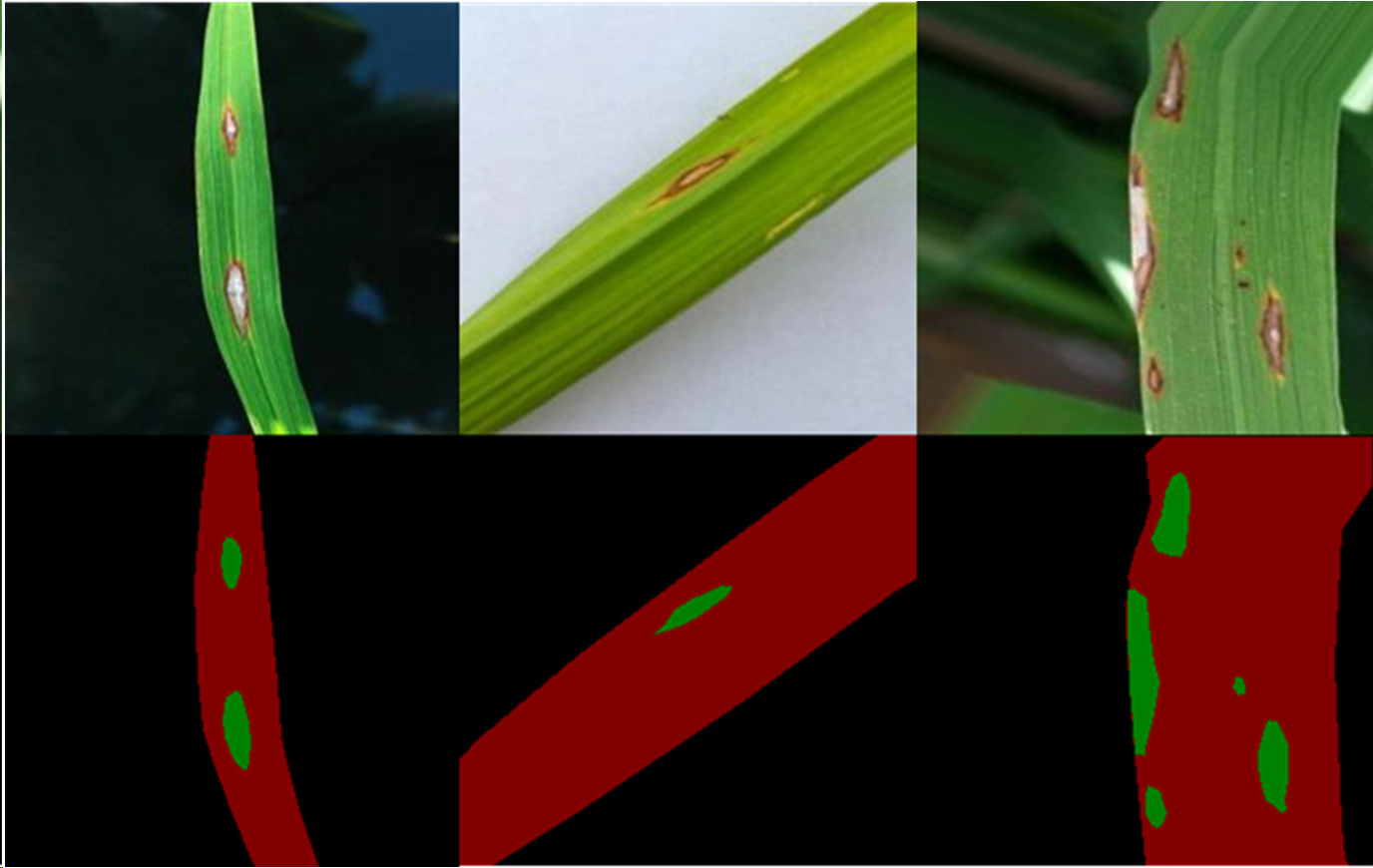


**Supplementary Figure 10.** Original images and labeled images: the black part means background, the red part means healthy rice leaf, while the green part means rice disease spot. (Reprinted from ref. [Feng et al., 2022a ] under the terms of the Creative Commons CC-BY license.)


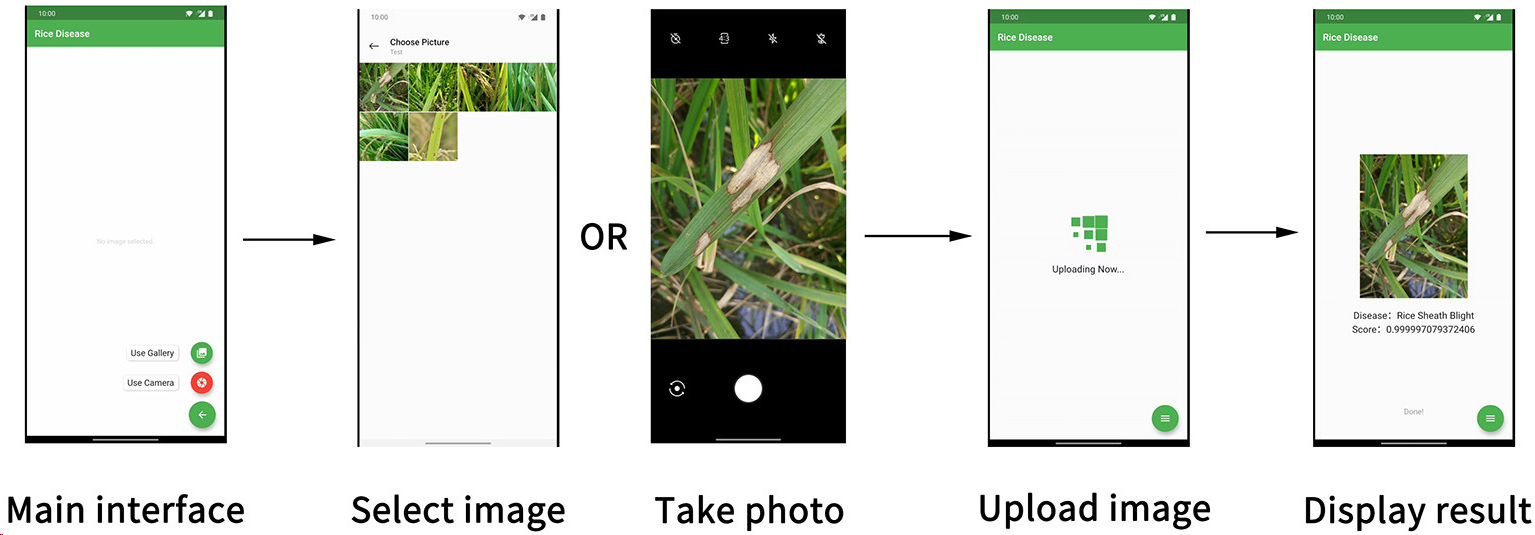


**Supplementary Figure 11.** The interface of the rice disease recognition APP. (Reprinted from ref. [Deng et al., 2021] under the terms of the Creative Commons CC-BY license.)


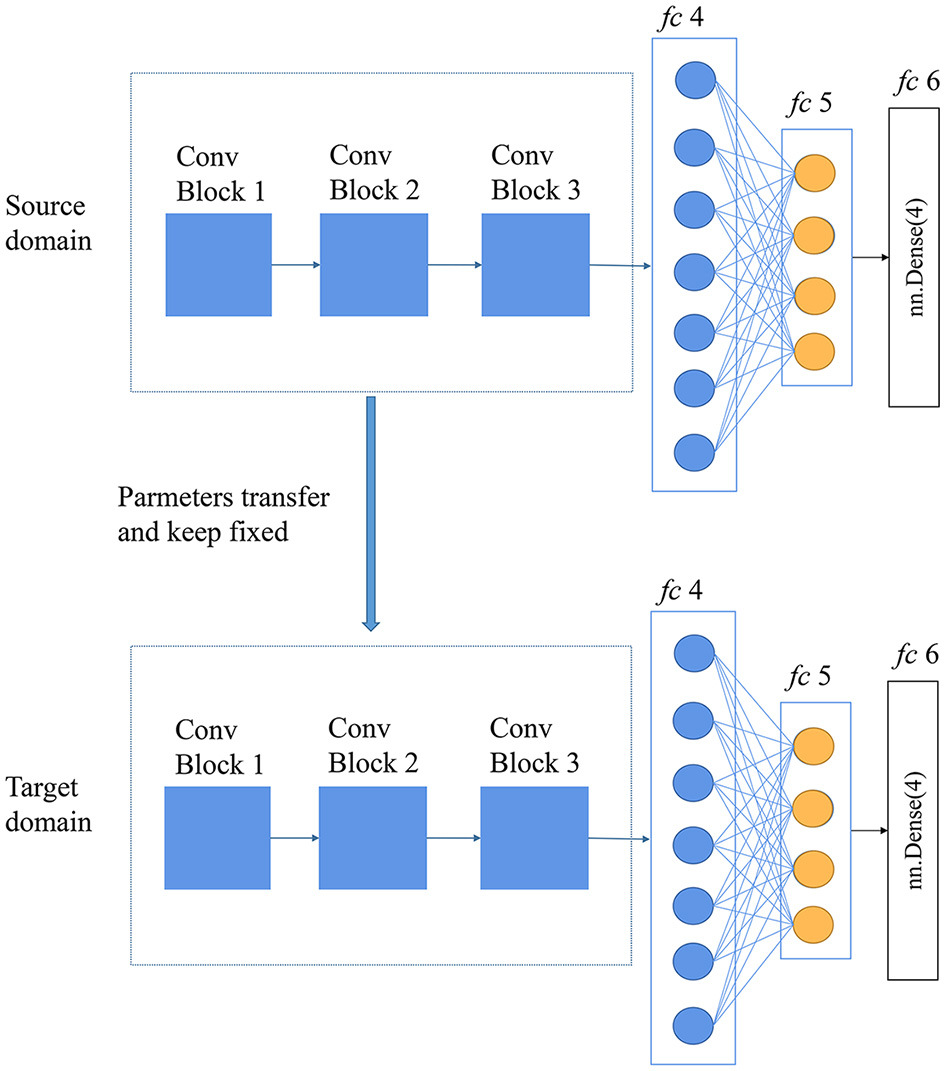


**Supplementary Figure 12**. Schematic diagram of the fine-tuning algorithm. (Reprinted from ref. [Feng et al., 2021a ] under the terms of the Creative Commons CC-BY license.)

## Supplementary Tables

Not applicable.
